# Mammalian Cell-Based Immunoassay for Detection of Viable Bacterial Pathogens

**DOI:** 10.3389/fmicb.2020.575615

**Published:** 2020-11-23

**Authors:** Luping Xu, Xingjian Bai, Shivendra Tenguria, Yi Liu, Rishi Drolia, Arun K. Bhunia

**Affiliations:** ^1^Molecular Food Microbiology Laboratory, Department of Food Science, Purdue University, West Lafayette, IN, United States; ^2^Purdue Institute of Inflammation, Immunology and Infectious Disease, Purdue University, West Lafayette, IN, United States; ^3^Department of Comparative Pathobiology, Purdue University, West Lafayette, IN, United States

**Keywords:** immunoassay, poultry, mammalian cells, *Salmonella*, detection, MaCIA, cell-based sensor, stress

## Abstract

Rapid detection of live pathogens is of paramount importance to ensure food safety. At present, nucleic acid-based polymerase chain reaction and antibody-based lateral flow assays are the primary methods of choice for rapid detection, but these are prone to interference from inhibitors, and resident microbes. Moreover, the positive results may neither assure virulence potential nor viability of the analyte. In contrast, the mammalian cell-based assay detects pathogen interaction with the host cells and is responsive to only live pathogens, but the short shelf-life of the mammalian cells is the major impediment for its widespread application. An innovative approach to prolong the shelf-life of mammalian cells by using formalin was undertaken. Formalin (4% formaldehyde)-fixed human ileocecal adenocarcinoma cell line, HCT-8 on 24-well tissue culture plates was used for the capture of viable pathogens while an antibody was used for specific detection. The specificity of the Mammalian Cell-based ImmunoAssay (MaCIA) was validated with *Salmonella enterica* serovar Enteritidis and Typhimurium as model pathogens and further confirmed against a panel of 15 *S.* Enteritidis strains, 8 *S*. Typhimurium, 11 other *Salmonella* serovars, and 14 non-*Salmonella* spp. The total detection time (sample-to-result) of MaCIA with artificially inoculated ground chicken, eggs, milk, and cake mix at 1–10 CFU/25 g was 16–21 h using a traditional enrichment set up but the detection time was shortened to 10–12 h using direct on-cell (MaCIA) enrichment. Formalin-fixed stable cell monolayers in MaCIA provide longer shelf-life (at least 14 weeks) for possible point-of-need deployment and multi-sample testing on a single plate.

## Introduction

Pathogen interaction with the host cells is the crucial first step for initiating infection (Finlay and Falkow, 1997; Kline et al., 2009), and harnessing such interaction may yield a robust detection platform not only to assess pathogenic potential but also its viability. Mammalian cell-based biosensors (CBBs) exploit host-pathogen interactions including pathogen adhesion, activation of host cell signaling events, cell-cycle arrest, apoptosis, and/or cytotoxicity (Banerjee and Bhunia, 2009). The ability to detect host-pathogen interaction makes CBB a functionality test, thus sets it apart from other conventional methods (Banerjee and Bhunia, 2009). A common approach to monitoring such interaction is to measure the cytotoxic effects of the analytes on mammalian cells. Gray et al. (2005) used lymphocyte (Ped-2E9)-based cytotoxicity assay to detect toxin produced by *Bacillus cereus*. Later, this cell line was used in a collagen-encapsulated 3-D platform to detect *Listeria monocytogenes* cells and its toxins (Banerjee et al., 2008) and several other toxin-secreting foodborne pathogens (Banerjee and Bhunia, 2010). Most recently, a 3-D Vero cell-platform was made to screen Shiga-toxin producing *Escherichia coli* (STEC) by measuring lactate dehydrogenase (LDH) release (To and Bhunia, 2019). Although these studies demonstrate the versatility of CBBs in detecting foodborne pathogens and toxins, the specificity of CBBs cannot be guaranteed when the detection solely relies on cytotoxicity measurement because cytosolic proteins/enzymes could be released from cells in response to more than one type of triggers. Furthermore, researchers have pointed out the short-comings of the practical applicability of CBBs due to the short shelf-life and the requirement for stringent growth conditions of mammalian cells outside a controlled laboratory environment (Bhunia et al., 1995; Banerjee et al., 2007; Banerjee and Bhunia, 2009; Ye et al., 2019). Thus, novel approaches for developing CBBs with higher specificity and longer shelf-life are in continued demand.

Pathogen detection is categorized into three basic types: culture-based, immunological, and nucleic acid-based (Bhunia, 2014; Lee et al., 2015; Bell et al., 2016; Schlaberg et al., 2017; Ricke et al., 2018; Rajapaksha et al., 2019). The detection time for the culture-based method is usually 4–7 days. Immunological and nucleic acid-based PCR methods are faster, but the inherent inability to assess the viability or the pathogenic potential of the target microorganisms is of concern (Bhunia, 2014; Kasturi and Drgon, 2017; Ricke et al., 2018). Moreover, these methods are often prone to interferences from sample inhibitors and resident microflora. Alternative detection methods that are faster, user-friendly, and accurate are in high demand (Bhunia, 2014). Therefore, CBBs have been proposed to serve as a reliable tool for the rapid screening of viable pathogens or active toxins in foods (Ngamwongsatit et al., 2008; Banerjee and Bhunia, 2009; Bhunia, 2011; Ye et al., 2019; To et al., 2020). However, maintaining the viability of mammalian cells outside the laboratory environment is a major challenge thus limits CBB’s utility in routine foodborne pathogen testing (Bhunia et al., 1995; Banerjee et al., 2007; Banerjee and Bhunia, 2009; Ye et al., 2019).

In this study, we took an innovative approach and developed a shelf-stable Mammalian Cell-based ImmunoAssay (MaCIA) platform for the detection of live pathogenic bacteria. Shelf-life of MaCIA was prolonged by fixing the mammalian cells in formalin (4% formaldehyde) which is a common practice in histology and tissue imaging to preserve the cells by preventing protein degradation (Eltoum et al., 2001). Furthermore, instead of measuring cytotoxicity, we took advantage of the adhesion ability of enteric pathogens to the intestinal cells followed by antibody-based assay for specific detection of the adhered target pathogens. Adhesion to the epithelial cells is the crucial first step for enteric pathogens (Kline et al., 2009; Dos Reis and Horn, 2010). For example, *L. monocytogenes* binds to Hsp60 and E-cadherin on the epithelial cell surface through *Listeria* adhesion protein (LAP) and Internalin A (InlA), respectively to initiate adhesion, invasion, translocation, and systemic spread during the intestinal phase of infection (Drolia et al., 2018; Drolia and Bhunia, 2019). Enterohaemorrhagic *E. coli* employs intimin, fimbrial proteins, flagella, and autotransporter proteins to attach to the host cells at different stages of its life cycle during infection (McWilliams and Torres, 2014). Likewise, *Salmonella enterica* utilizes multiple fimbrial adhesins, such as type 1 fimbriae (T1F) and long polar fimbriae (Lpf), and several autotransporter adhesins, such as ShdA and MisL, to promote adhesion to D-mannose receptors on M cells in Peyer’s Patches and assist colonization in the intestine (Bäumler et al., 1996; Wagner and Hensel, 2011; Bhunia, 2018; Kolenda et al., 2019). Therefore, detecting only adhered pathogens using antibodies is a rational approach. We chose human ileocecal adenocarcinoma cell line, HCT-8, as the target cells for building MaCIA platform on 24-well tissue culture plates. HCT-8 is one of the commonly used model cell lines to study the adhesion of enteric pathogens (McKee and O’Brien, 1995; Dibao-Dina et al., 2015; Hu and Wai, 2017). Unlike other cell lines used, HCT-8 cells can form a fully confluent monolayer in only 5 days.

The objective of this study was to develop a shelf-stable MaCIA platform for the rapid detection of viable bacterial pathogens and to validate its performance using *Salmonella enterica* serovar Enteritidis as a model foodborne pathogen.

*Salmonella enterica* is a major foodborne pathogen of global public health concern. Meat, poultry, eggs, nuts, fruits, and vegetables are common vehicles for *Salmonella* transmission. Each year, *Salmonella* infections contribute to 1.3 billion cases of gastroenteritis and 3 million deaths worldwide (Kirk et al., 2015) and 1.35 million cases, 26,500 hospitalizations, and 420 deaths in the United States (CDC, 2020). Among *Salmonella* serovars, *Salmonella enterica* serovar Enteritidis is one of the most prevalent serovars in the United States. The Centers for Disease Control and Prevention (CDC) has reported eight major outbreaks between 2006 and 2018 resulting in about 4,000 cases (CDC, 2018). In a survey of salmonellosis outbreaks (total 2,447) in the United States between 1998 and 2015, *S*. Enteritidis (29.1%) was reported to be the most common serovar followed by *S*. Typhimurium (12.6%), *S*. Newport (7.6%), and others (Snyder et al., 2019). The frequent occurrence of food-associated *S.* Enteritidis outbreaks with the high number of infections was the motivation for developing a mammalian cell-based functional bioassay for the detection of *S.* Enteritidis.

The initial study involved screening of MaCIA with a panel of food-associated bacterial cultures (Table 1) in confirming the specificity and the limit of detection (LOD) from artificially inoculated food samples. Next, the performance of MaCIA was validated using the US Department of Agriculture (USDA-FSIS, 2013) and the Food and Drug Administration (FDA, 2001) reference methods. “On-cell enrichment” and “one-step antibody probing methods” of MaCIA were also explored to reduce the assay steps and total detection time. Overall, the data showed that MaCIA could detect viable *S.* Enteritidis (1–10 CFU/25 g) in ground chicken, shelled eggs, whole milk, and cake mix using a traditional enrichment set up, but the detection time was shortened to 10–12 h using direct on-cell (MaCIA) enrichment. We also demonstrated the versatility of MaCIA by using a commercial anti-*Salmonella* reporter antibody for the detection of *S*. Typhimurium. Formalin-fixed cells in the MaCIA platform permits a longer shelf life (at least 14-week at 4°C), minimum on-site maintenance care, and a stable cell monolayer for point-of-need deployment.

**TABLE 1:** Specificity of mammalian cell-based immunoassay(MaCIA) platform tested against *Salmonella* and non-*Salmonella* spp.

Bacteria	CFU/Well	MaCIA Result*			
		
		mAb-2F11		mAb-F68C	
			
		Abs_450nm_ ± SD	Result	Abs_450nm_ ± SD	Result
***Salmonella enterica* serovars**					
Enteritidis PT21	2.0–13 × 10^7^	0.95 ± 0.08	+	0.10 ± 0.01	-
Enteritidis 13ENT1344	2.9 × 10^7^	1.13 ± 0.16	+	NT	NT
Enteritidis 13ENT1374	2.8–3.3 × 10^7^	0.91 ± 0.15	+	0.09 ± 0.00	-
Enteritidis 13ENT1376	2.0 × 10^7^	1.06 ± 0.03	+	NT	NT
Enteritidis 13ENT1375	3.1 × 10^7^	1.07 ± 0.15	+	NT	NT
Enteritidis 13ENT1032	2.2 × 10^7^	1.08 ± 0.25	+	NT	NT
Enteritidis PT1	2.8 × 10^7^	1.19 ± 0.04	+	NT	NT
Enteritidis PT4	2.0 × 10^7^	1.17 ± 0.06	+	NT	NT
Enteritidis PT6	1.8 × 10^7^	1.41 ± 0.04	+	NT	NT
Enteritidis PT7	7.5 × 10^6^	0.70 ± 0.06	+	NT	NT
Enteritidis PT8	1.4 × 10^7^	1.42 ± 0.06	+	NT	NT
Enteritidis PT13a	1.5 × 10^7^	0.74 ± 0.02	+	NT	NT
Enteritidis PT13	1.1 × 10^7^	0.90 ± 0.06	+	NT	NT
Enteritidis PT14b	1.3 × 10^7^	1.05 ± 0.04	+	NT	NT
Enteritidis PT28	1.1 × 10^7^	0.53 ± 0.05	+	NT	NT
Typhimurium 13ENT906	6.7–8.8 × 10^7^	0.33 ± 0.03	-	1.14 ± 0.06	+
Typhimurium NOS12	4.0 × 10^7^	0.33 ± 0.03	-	0.98 ± 0.04	+
Typhimurium NOS3	3.3 × 10^8^	NT	NT	0.80 ± 0.04	+
Typhimurium NOS10	1.3 × 10^8^	NT	NT	0.90 ± 0.12	+
Typhimurium NOS2	6.7 × 10^8^	NT	NT	0.73 ± 0.08	+
Typhimurium NOS4	6.7 × 10^8^	NT	NT	0.89 ± 0.04	+
Typhimurium NOS1	3.3 × 10^7^	NT	NT	0.84 ± 0.06	+
Typhimurium ST1	3.8 × 10^6^	0.13 ± 0.02	-	NT	NT
Newport 13ENT1060	2.3–23 × 10^7^	0.32 ± 0.04	-	0.13 ± 0.01	-
Braenderup 12ENT1138	6.3 × 10^7^	0.33 ± 0.03	-	NT	NT
Agona 12ENT1356	2.7–13 × 10^8^	0.32 ± 0.02	-	0.09 ± 0.01	-
Hadar 13ENT979	4.3 × 10^7^	0.27 ± 0.02	-	NT	NT
Paratyphi 11J85	2.4 × 10^7^	0.27 ± 0.05	-	NT	NT
Heidelberg 18ENT1418	4.0 × 10^7^	0.29 ± 0.04	-	NT	NT
Saintpaul 13ENT1045	5.0 × 10^7^	0.30 ± 0.04	-	NT	NT
Javiana 13ENT86F	0.4–2.7 × 10^8^	0.38 ± 0.10	-	0.14 ± 0.07	-
Infantis 13ENT866	2.0 × 10^7^	0.32 ± 0.02	-	NT	NT
Bareilly 12ENT1164	0.1–4.0 × 10^8^	0.32 ± 0.09	-	NT	NT
Pullorum DUP-PVUII 1006	1.9 × 10^7^	0.34 ± 0.04	-	NT	NT
**Miscellaneous**					
*Listeria monocytogenes* F4244	0.5–1.6 × 10^8^	0.27 ± 0.03	-	0.13 ± 0.01	-
*L. innocua* F4248	5.0 × 10^7^	0.27 ± 0.03	-	NT	NT
*Escherichia coli* O157:H7 EDL933	0.4–1.3 × 10^8^	0.26 ± 0.03	-	0.08 ± 0.04	-
*Hafnia alvei* 18066	3.3–6.3 × 10^7^	0.28 ± 0.03	-	0.15 ± 0.02	-
*Citrobacter freundii* ATCC8090	0.3–1.0 × 10^8^	0.29 ± 0.02	-	0.13 ± 0.01	-
*Citrobacter freundii* ATCC43864	0.4–3.3 × 10^7^	0.11 ± 0.00	-	0.11 ± 0.01	-
*Citrobacter freundii* ATCC3624	0.3–1.3 × 10^8^	0.13 ± 0.01	-	0.12 ± 0.02	-
*Serratia marcescens* ATCC8100	0.6–5.3 × 10^7^	0.33 ± 0.02	-	0.12 ± 0.01	-
*Serratia marcescens* ATCC43862	0.1–1.0 × 10^8^	0.11 ± 0.01	-	0.13 ± 0.01	-
*Serratia marcescens* B-2544	0.6–3.3 × 10^7^	0.13 ± 0.01	-	0.11 ± 0.02	-
*Pseudomonas aeruginosa* PRI99	2.25 × 10^7^	0.24 ± 0.02	-	NT	NT
*Proteus mirabilis* B-3402	0.7–6.7 × 10^8^	0.11 ± 0.01	-	0.11+0.01	-
*Proteus vulgaris* DUP-10086	0.4–4.0 × 10^8^	0.11 ± 0.01	-	0.10 ± 0.02	-
*Klebsiella pneumoniae* B-41958	6.7 × 10^6^	0.10 ± 0.01	-	0.13 ± 0.01	-

*Values are from four independent replicates; Results (±) are decided by comparing to the negative control in each experiment. Values that are significantly different (*P* < 0.001) from the negative control in each experiment are regarded as +; NT, not tested.

## Results

### Development of MaCIA (Mammalian Cell-Based ImmunoAssay) Platform

**FIGURE 1:**
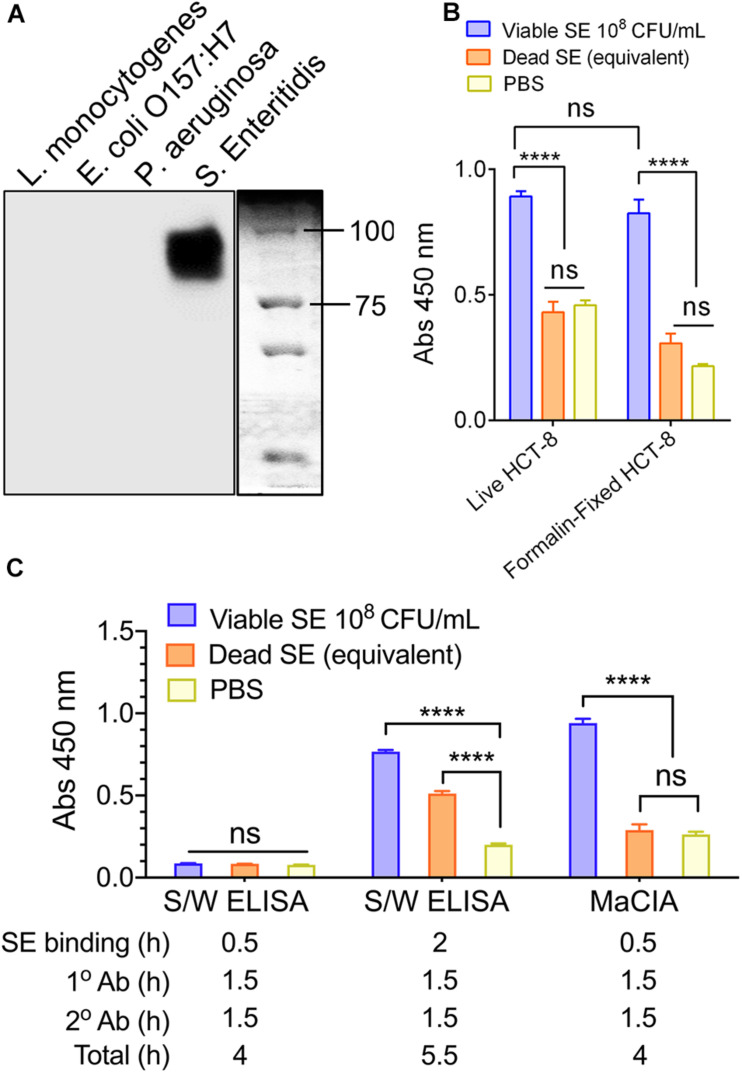
Mammalian cell-based immunoassay (MaCIA) development. **(A)** Western blot showing the reaction of mAb-2F11 to *Salmonella enterica* serovar Enteritidis PT21 but not to *L. monocytogenes* F4244, *E. coli* O157:H7 EDL933 and *Pseudomonas aeruginosa* PRI99. **(B)** MaCIA analysis with live (Live HCT-8) and formalin-fixed (Formalin-fixed HCT-8) HCT-8 cell. **(C)** Comparison of MaCIA with sandwich ELISA (S/W ELISA). Error bars represent the standard error of the mean (SEM). ****P < 0.0001; ns, no significance. Cut-off for positive: *P* < 0.001. SE, *S*. enteritidis; Ab, antibody.

The MaCIA platform was built on a 24-well tissue culture plate, and it consisted of two steps: fixation of mammalian cells and immunoassay for specific detection of adherent target pathogens. We used the formalin-fixed HCT-8 cell line for *Salmonella* adhesion/capture (30 min) and anti-*S.* Enteritidis monoclonal antibody, mAb-2F11 (Masi and Zawistowski, 1995), or anti-*Salmonella* mAb-F68C (Thermo Fisher Scientific; 1.5 h), horseradish peroxidase (HRP)-conjugated second antibody and a substrate for color development (1.5 h). The mAb-2F11 is highly specific for *S.* Enteritidis (Masi and Zawistowski, 1995; Jaradat et al., 2004), and the Western blot analysis confirmed its specificity without showing any reaction with bands from whole-cell preparations of *L. monocytogenes*, *E. coli* O157:H7 or *Pseudomonas aeruginosa* (Figure 1A).

To fix mammalian cells on the MaCIA platform, HCT-8 cell monolayers in 24 well-plates were treated with a 4% formaldehyde solution for 10 min, followed by three sequential wash using phosphate-buffered saline (PBS, 0.1 M, pH 7). Initially, the performance of formalin-fixed MaCIA was compared with a live cell-based MaCIA platform to detect *S*. Enteritidis PT21 that was incubated for 30 min at 37°C. Remarkably, both assay configurations showed strong positive signals toward viable *S*. Enteritidis, which was significantly (*P* < 0.0001) higher than the equivalent amounts of dead *S*. Enteritidis cells (verified by plating) and the negative control (PBS) (Figure 1B).

The performance of MaCIA was also compared with traditional sandwich ELISA where mAb-2F11 was used as capture and anti-*Salmonella* pAb-3238 (Abdelhaseib et al., 2016) was used as the reporter. MaCIA gave positive results when tested with viable *S*. Enteritidis cells (1 × 10^8^ CFU/mL), which is significantly higher (*P* < 0.0001) than that of the equivalent numbers of dead cells or the PBS control. On the other hand, both viable and dead *S*. Enteritidis cells showed positive signals with sandwich ELISA, though the signals for viable cells were slightly higher than those of the dead cells (Figure 1C). Furthermore, the total detection time (after addition of bacteria to the wells of assay plates) required for sandwich ELISA was 5.5 h, while 4 h for MaCIA (Figure 1C).

### Specificity of the MaCIA Platform

**FIGURE 2:**
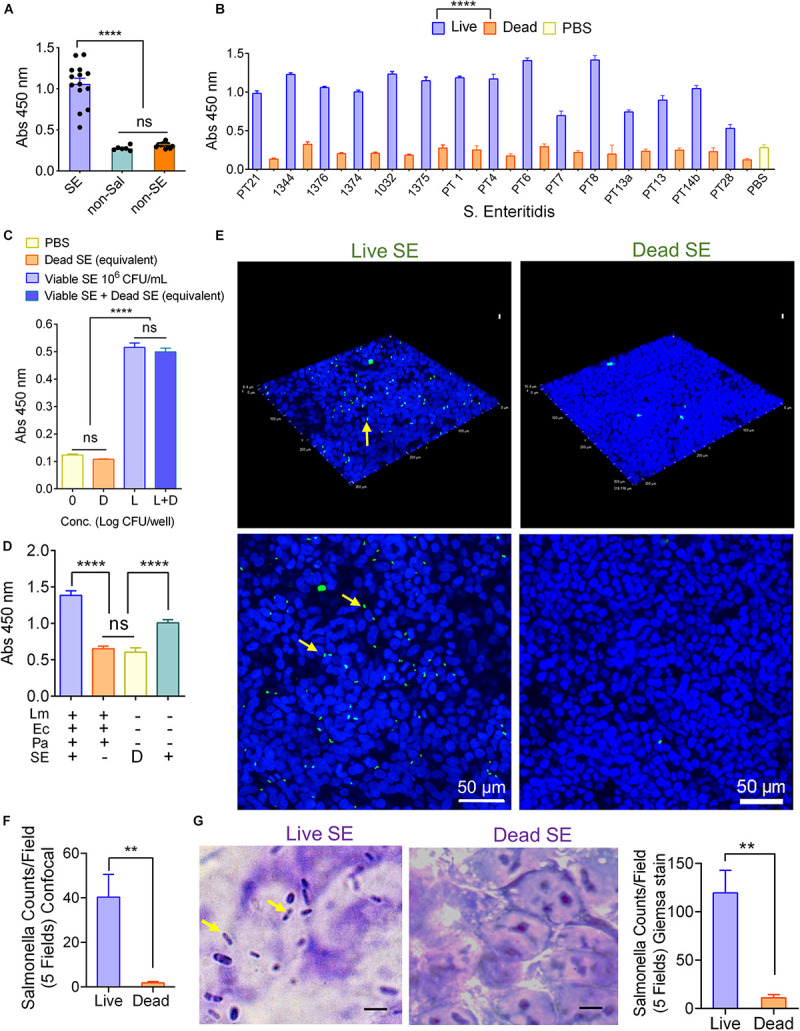
Mammalian cell-based immunoassay specificity. MaCIA reaction with 15 *Salmonella* Enteritidis strains (SE), 12 non-SE and 7 non-*Salmonella* bacteria **(A)**, with viable and dead *S*. Enteritidis serovars **(B)**, to viable *S.* Enteritidis in the presence of the equivalent amount of dead *S.* Enteritidis **(C)**, and *S*. Enteritidis PT21 in the presence of other bacteria (Lm, *L. monocytogenes* F4244; Ec, *E. coli* EDL933 and Pa, *Pseudomonas aeruginosa* PRI99). L: live SE; D: Dead SE **(D)**. Confocal image and Giemsa staining analyses of adhesion of live (Live SE) and dead (Dead SE) *S*. Enteritidis PT21 to formalin-fixed HCT-8 cells; **(E)** Z-stack of the scanned images, **(F)** total bacterial counts per five fields for confocal images. Blue: nucleus, green: *S*. Enteritidis, **(G)** Giemsa stained images showing adhesion of live (Live SE) but not dead (Dead SE) *S*. Enteritidis PT21 to formalin-fixed HCT-8 cells. Rod-shaped dark blue, *S.* Enteritidis (arrows); purple, nucleus; Bar graph showing bacterial counts per field from five fields. Error bars represent SEM. ****P < 0.0001; **P < 0.01; ns, no significance. Cut-off for positive: *P* < 0.001. Scale bar = 5 μm.

**FIGURE 3:**
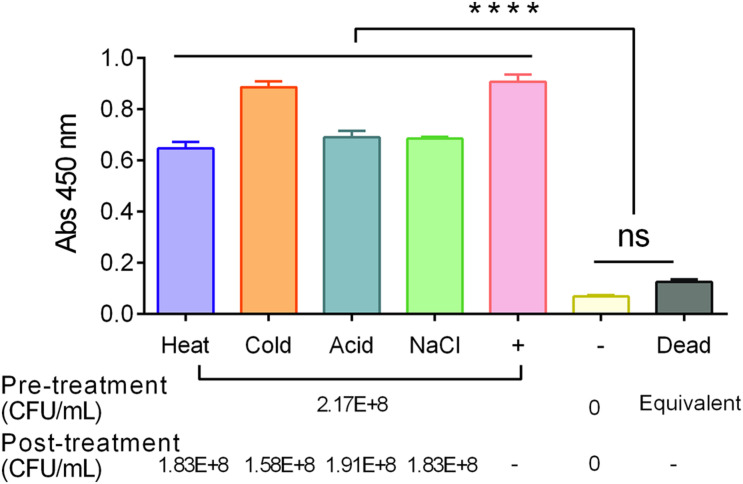
Detection of stress-exposed *S.* Enteritidis PT21 using MaCIA. Bacteria were exposed to heat (45°C), cold (4°C), acidic pH (4.5) and NaCl (5.5%) for 3 h before analysis. +, Positive control (bacteria without any stress exposure); –, No bacteria; dead, heat-killed *S*. Enteritidis cells. Error bars represent SEM. ****P < 0.0001; ns, no significance.

Next, the specificity of the MaCIA was determined by testing a panel of 15 *S*. Enteritidis strains, eight *S*. Typhimurium strains, 11 other *Salmonella* serovars, and 14 non-*Salmonella* spp. at ∼ 1 × 10^6^ to 1 × 10^7^ CFU/mL each. The data showed that MaCIA was highly specific toward all tested viable strains of *S.* Enteritidis or *S*. Typhimurium serovars depending on the reporter antibody used and the signals were significantly (*P* < 0.001) higher than the signals obtained for other *Salmonella* serovars or non-*Salmonella* species (Figures 2A,B and Table 1). Thus, any sample showing a significantly higher signal (*P* < 0.001) than the negative control was considered positive. Furthermore, samples containing live *S*. Enteritidis cells gave significantly (*P* < 0.0001) higher absorbance values (signals) than that of the values obtained for dead cells or the PBS control (Figure 2B). The specificity of MaCIA toward viable cells was not affected when tested against a mixture containing equal amounts of viable and dead *S.* Enteritidis cells (Figure 2C), and non-*S*. Enteritidis bacteria (Figure 2D). Immuno-stained confocal images, the Z-stacking (three-dimensional image), and Giemsa stain images confirmed increased adhesion of viable *S*. Enteritidis cells to HCT-8 cells than that of the dead *S*. Enteritidis cells (Figures 2E–G). Confocal imaging further revealed the absence of non-specific binding of mAb-2F11 to the HCT-8 cell monolayer (Figures 2E,F). Furthermore, MaCIA successfully detected *S*. Enteritidis cells when exposed to various stressors for 3 h (Hahm and Bhunia, 2006) including cold (4°C), heat (45°C), acidic pH (4.5), and 5.5% NaCl (Figure 3).

### Detection Sensitivity of MaCIA

**FIGURE 4:**
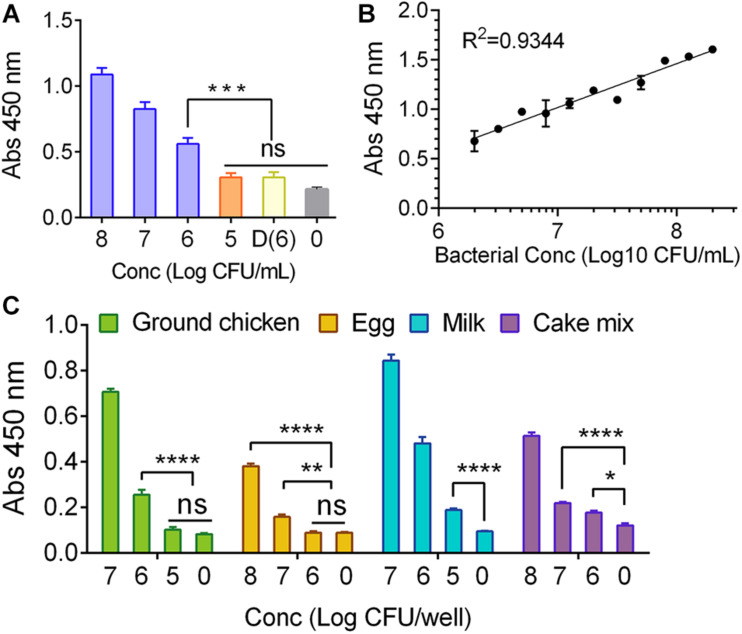
Assay sensitivity for MaCIA. **(A)** Analysis of limit of detection (LOD) of MaCIA against *S.* Enteritidis PT21 at 1 × 10^5^ CFU/mL to 1 × 10^8^ CFU/ml suspended in PBS; and **(B)** corresponding correlation coefficient of absorbance and bacterial concentration. **(C)** Analyses of LOD of MaCIA when *S.* Enteritidis PT21 was suspended in different food matrices. 0, no bacteria; D(6), dead *S.* Enteritidis PT21 at 1 × 10^6^ cells/ml. In all figures, samples with higher concentrations were also significantly (*P* < 0.001) different than the dead samples and negative control. Error bars represent SEM. ****P < 0.0001; ***P < 0.001; **P < 0.01; *P < 0.05; ns, no significance. Cut-off for positive: *P* < 0.001.

To determine assay sensitivity, *S*. Enteritidis cells were serially diluted using either PBS or ground chicken suspended in buffered peptone water (BPW) and added to the wells containing formalin-fixed HCT-8 cell monolayers (30-min post-fixation). After a 30-min incubation at 37°C with test samples, the monolayers were washed, probed with mAb-2F11, and the color was developed. An initial bacterial concentration of 1 × 10^6^ to 1 × 10^8^ CFU/mL showed significantly (*P* < 0.001) higher signal than the wells containing 1 × 10^5^ CFU/mL or dead cells (1 × 10^6^ cells) suspended in PBS (Figure 4A) or ground chicken slurry (Supplementary Figure 1) and the absorbance values showed strong correlation (*R*^2^ = 0.9344) with *S.* Enteritidis cell numbers (1 × 10^6^ CFU/mL to 1 × 10^8^ CFU/mL) (Figure 4B). Furthermore, MaCIA also showed a similar concentration-dependent rise in signals when bacteria were suspended in ground chicken, liquid egg, milk, and cake mix slurry (Figure 4C). However, the detection sensitivity varied depending on the food matrix tested. In milk, the detection limit was determined to be 1 × 10^5^ CFU/mL while in ground chicken, 1 × 10^6^ CFU/mL, in cake mix, 1 × 10^7^ CFU/mL, and in egg, 1 × 10^8^ CFU/mL (Figure 4C). These results indicate that assay sensitivity for MaCIA for the detection of *S*. Enteritidis varies from 1 × 10^5^ CFU/mL to 1 × 10^8^ CFU/mL depending on the food matrix tested.

### Further Optimization of MaCIA

#### One-Step Antibody Probing Method

**FIGURE 5:**
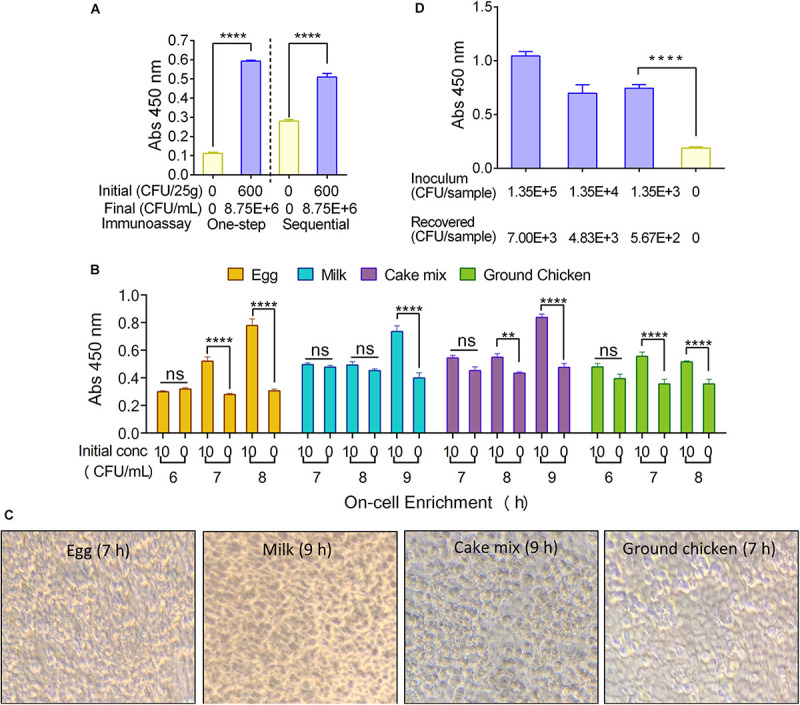
Mammalian cell-based immunoassay assay optimization. **(A)** One-step antibody probing vs sequential antibody probing against a bacterial cell concentration of 8.75 × 10^6^ CFU/ml of *S*. Enteritidis. **(B)** Analysis of time (h) required for positive MaCIA result during on-cell enrichment of *S.* Enteritidis PT21 (∼10 CFU/mL) inoculated into different food products. **(C)** Light microscopic images of formalin-fixed HCT-8 cell monolayers after on-cell enrichment for 7–9 h. Magnification (400×). **(D)** MaCIA analysis of skin swab samples after on-cell enrichment (7 h). Samples with higher concentrations were also significantly (*P* < 0.001) different than the negative control. Error bars represent SEM. ****P < 0.0001; **P < 0.01; ns, no significance. Cut-off for positive: *P* < 0.001.

To shorten the detection time, we explored if a one-step antibody probing approach is feasible. Ground chicken was inoculated with *S*. Enteritidis at 6 × 10^2^ CFU/25 g in a stomacher bag. After 10-h enrichment at 37°C, the enriched chicken samples (1 mL) were added to the fixed HCT-8 cell monolayer for 30 min, followed by PBS wash (3 times). The cell monolayers were probed with an antibody cocktail that contained both primary (mAb-2F11) and secondary (anti-mouse HRP-conjugated IgG) antibodies, followed by the colorimetric substrate. Data showed that the signal obtained from the one-step antibody probing was comparable to the results when the sequential antibody probing method was used (Figure 5A). This experiment indicates that one-step antibody probing is equally effective as the sequential antibody probing method, thus shortening the assay time by 2.5 h.

#### On-Cell Food Sample Enrichment

Direct on-cell (MaCIA platform) enrichment of test samples was pursued to simplify the assay procedure and to reduce the sample handling steps. *S*. Enteritidis inoculated food suspensions (with an initial inoculation of 10 CFU/mL) were directly added to the wells (1 mL/well) containing formalin-fixed HCT-8 cell monolayers and incubated at 37°C. The assay was performed after 6, 7, 8, and 9-h on-cell sample enrichment followed by sequential antibody probing (3 h). After 7-h on-cell enrichment, both ground chicken and egg samples gave positive results while the whole milk and cake mix needed 9-h enrichment to give positive results when compared with uninoculated food samples (Figure 5B). A similar result was obtained when the food samples were tested in a blinded fashion (Supplementary Figure 2). Total assay time (sample-to-result) for on-cell enrichment was estimated to be 10–12 h. Remarkably, the HCT-8 cell monolayers remained intact without any visible damage during on-cell enrichment (Figure 5C). Due to the limitation in the amount of sample volume (1 mL/well), that can be tested, the “on-cell enrichment” option is suitable only when the starting *S.* Enteritidis concentration is above 10 CFU/mL (2.5 × 10^3^ CFU/25 g); hence it may not be suitable for routine testing of bulk-food samples that may contain < 100 CFU/g.

We then examined if the on-cell enrichment set up is suitable for testing surface swab samples. Skin swabs from inoculated chicken thigh parts (1.35 × 10^3^ to 1.35 × 10^5^ CFU/50 cm^2^ at 4°C for 24 h) were resuspended in 1.1 mL of BPW, and 1 ml of each suspension was added to the wells of MaCIA. After 7-h on-cell enrichment followed by sequential immunoprobing (3 h), MaCIA generated significantly (*P* < 0.0001) higher signals than that of the values obtained from the negative control (swabbed suspension of the uninoculated sample) (Figure 5D). These data indicate that MaCIA is suitable for testing surface swab samples, and results can be obtained in less than 12 h.

### Comparison of MaCIA With the USDA/FDA Detection Methods

To compare the performance of MaCIA with USDA/FDA detection methods, *S*. Enteritidis inoculated food samples (ground chicken, egg, milk and cake mix held at 4°C for 24 h) were also tested in parallel using the US Department of Agriculture (USDA-FSIS, 2013) or Food and Drug Administration (FDA, 2001) reference method.

#### Growth Kinetics of *S*. Enteritidis in Different Foods

**FIGURE 6:**
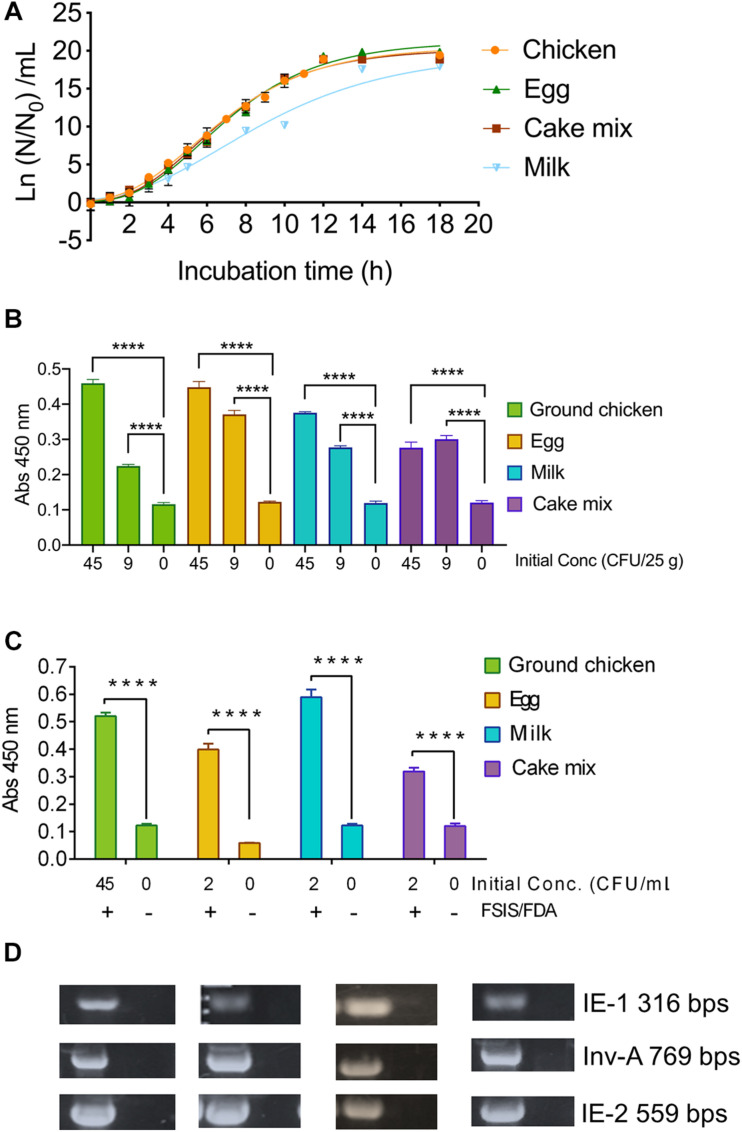
Mammalian cell-based immunoassay validation with inoculated food samples. **(A)** Growth curve of *S.* Enteritidis PT21 in various food products suspended in buffered-peptone water (BPW) at 37°C. Before growth analysis, inoculated food samples were held at 4°C for 24 h. The best-fit curves for *Salmonella* growth in different foods were generated by using the Gompertz model. *R*^2^ values of each fitted Gompertz curve are 0.99 (Chicken), 0.99 (Egg), 0.99 (cake mix) and 0.96 (Milk). N_0,_ initial *S.* Enteritidis concentration; N, *S.* Enteritidis concentration at the corresponding time point. MaCIA results of *S*. Enteritidis inoculated (at 0, 9, 45 CFU/25 g) **(B)** and at 0, 2, 45 CFU/mL **(C)** food samples after 14–19 h enrichment. **(D)** PCR confirmation of *S.* Enteritidis targeting *Salmonella* specific genes. Error bars represent SEM. ****P < 0.0001.

Freshly grown (37°C, 18 h) *S.* Enteritidis PT21 culture was inoculated (<10 CFU/ml) into 25 g of each ground chicken, egg, whole milk, or cake mix in 225 mL BPW in a stomacher bag (Seward Inc., Bohemia, NY, United States) and held at 4°C for 24 h. Inoculated food samples were then incubated at 37°C and bacterial counts were determined every 2-h intervals until 18 h. The growth data of *S.* Enteritidis in all tested food samples were fitted with the Gompertz model to generate a growth curve (Figure 6A). The *R*^2^ values of Gompertz fitted growth curves of *S.* Enteritidis PT21 in ground chicken, egg, whole milk, and cake mix were 0.99, 0.99, 0.96, and 0.99, respectively. Based on the Gompertz modeled growth curve equations, the lag phase duration (LPD) and exponential growth rate (EGR) were estimated to be 2.204–2.427 h and 0.767–0.934 log (CFU/mL)/h, respectively (Supplementary Table 1). Utilizing LPD, EGR, and the MaCIA detection limit data, we were able to estimate the required enrichment time for each food product, assuming the starting *S.* Enteritidis concentration is 1 CFU/25 g (Supplementary Table 1). The required enrichment time for ground chicken, egg, milk, and cake mix was estimated to be 14, 19, 16, and 16 h, respectively (Supplementary Table 1).

#### Sample-to-Result Time

To confirm the sample-to-result time, we inoculated the selected food samples with *S*. Enteritidis at 0, 9, or 45 CFU/25 g (Figure 6B) and 0, 2 or 45 CFU/mL (Figure 6C). After a specified enrichment period, we analyzed the samples using MaCIA. All *S*. Enteritidis-inoculated samples produced significantly higher signals (*P* < 0.001) than the uninoculated food samples (Figures 6B,C) even in the presence of background microflora (Supplementary Figure 3). The sample-to-result time was estimated to be 16–21 h. Analysis of food samples by the USDA-FSIS or FDA-BAM method followed by polymerase chain reaction (PCR) assay using three sets of primers targeting *invA*, IE-1, and IE-2 genes (Figure 6D) confirmed the presence of *S*. Enteritidis in these food samples. Note, the USDA method needed 72 h, while the FDA method needed 72–168 h to confirm the presence of *Salmonella* in the inoculated food samples.

### Formalin-Fixation Prolongs the Shelf-Life of MaCIA

**FIGURE 7:**
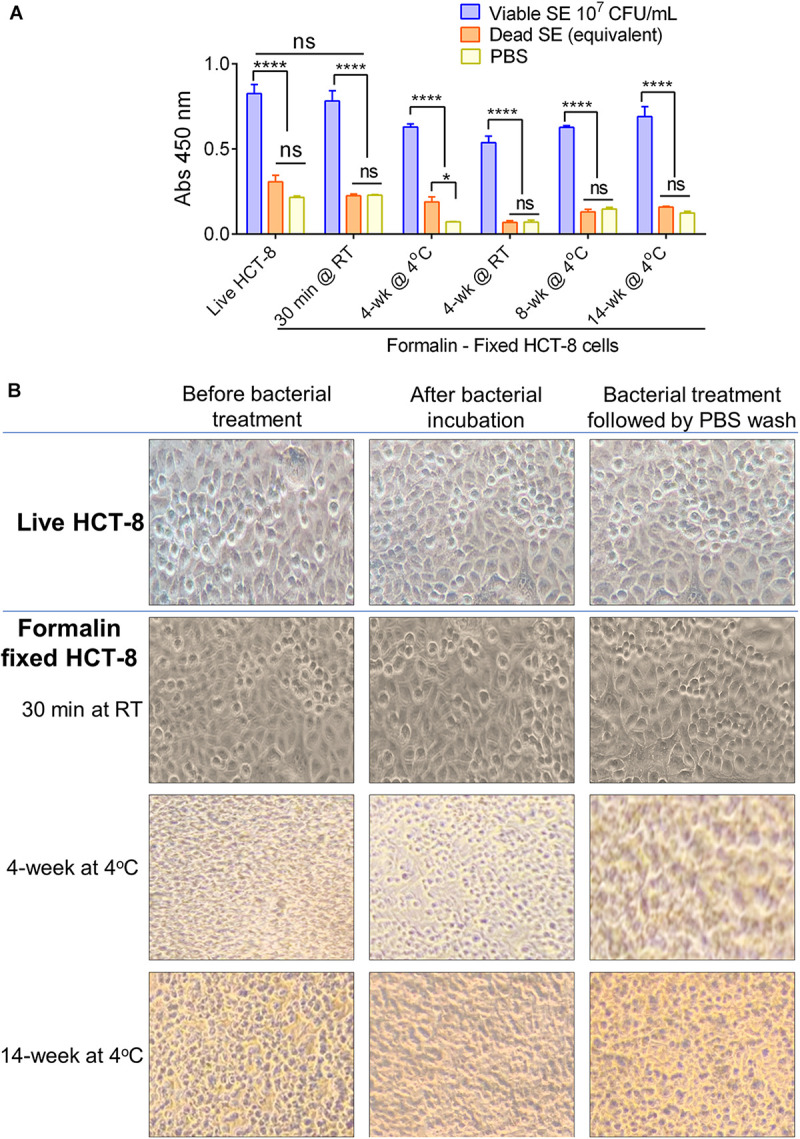
Performance of MaCIA after prolonged storage. **(A)** Comparison of MaCIA signals (absorbance reading) of *S*. Enteritidis cells (1 × 10^7^ cells/ml) originating from live HCT-8 and formalin-fixed HCT-8 cells stored at 4°C for 30 min to 14 weeks. **(B)** Light microscopic analysis of cell morphology of formalin-fixed HCT-8 cells stored up to 14 weeks. Panels showing intact cell morphology before bacterial treatment, after treatment, and after PBS wash. Magnification, 400×. Error bars represent SEM. ****P < 0.0001; ns, no significance.

The bottleneck for widespread use of cell-based sensors is its limited shelf-life. As we have demonstrated earlier (Figure 1B), the performance of MaCIA prepared with live HCT-8 cells is equally sensitive to the formalin-fixed HCT-8 cells (30 min after fixation). In this experiment, we investigated if the prolonged storage (4, 8, and 14 weeks at 4°C or 4 weeks at room temperature) of formalin-fixed HCT-8 cell would uphold MaCIA’s performance. Data showed that formalin-fixed HCT-8 cells stored for 4–12 weeks at 4°C generated comparable results to that of live HCT-8 cells when tested with viable *S.* Enteritidis PT21 at a concentration of 1 × 10^7^ CFU/ml and signals were significantly higher (*P* < 0.0001) than the signals generated by an equivalent amount of dead *S*. Enteritidis cells or the PBS control (no bacteria) (Figure 7A). The light microscopic photomicrographs further confirmed that the cell monolayer and the cellular morphology in formalin-fixed HCT-8 cells were unaffected after 14 weeks of storage at 4°C or even after bacterial exposure and the subsequent three PBS wash (Figure 7B). These results indicate that formalin fixation was able to prolong the shelf-life of HCT-8 cells up to 14 weeks without affecting their performance, thus showing a promising application of the MaCIA for point-of-need deployment.

## Discussion

The conventional culture-based detection methods (sample-to-result) take 4–7 days to obtain the results (FDA, 2001; USDA-FSIS, 2013; Bell et al., 2016), and the so-called rapid methods take at least 24–48 h (Bhunia, 2014; Lee et al., 2015; Ricke et al., 2018; Rajapaksha et al., 2019). This is a major inconvenience for the food industries since some foods have a limited shelf-life. Furthermore, holding of products until the microbiological safety assessment can also increase the cost of storage. Therefore, products are released into the supply chain even before obtaining test results. Such practice is very costly, resulting in more than hundreds of recalls each year and the loss of millions of pounds of food (Buzby et al., 2014; Elkhishin et al., 2017). Therefore, rapid, accurate, and user-friendly viable pathogen detection tools are in high demand to lower recalls, reduce food waste and financial loss, and prevent foodborne outbreaks.

Mammalian cell-based assays are highly attractive functional screening tools to assess the presence of viable pathogens or active toxins in near-real-time (Bhunia, 2011, 2014; To et al., 2020). CBB monitors host-hazard interaction (Banerjee and Bhunia, 2009); therefore, non-pathogenic, non-hazardous, dead, or non-toxic agents do not yield false results. However, the major drawback is its short self-life, i.e., the mammalian cells may not survive on the sensor platform for a prolonged period without the proper growth conditions. Mammalian cells have stringent requirements for specialized growth media and growth conditions for survival, such as temperature and CO_2_-controlled humidified environment. Limited self-life of cells is a monumental deterrent for CBB’s widespread application affecting its deployment for point-of-need use. To overcome the limitation, we employed formalin (4% formaldehyde) to preserve the functionality of the mammalian cells. We used the human ileocecal cell line, HCT-8, as our model cell line, which maintained its functionality after formalin-fixation, at least for 14 weeks at 4°C. The fixed HCT-8 cells showed selective interaction with viable or even stress-exposed *Salmonella*, while dead cells had negligible or no interaction at all (Figures 1–3). Further specificity of the assay was accomplished by immunoprobing the adhered bacterial cells using a specific antibody. The MaCIA was found to be highly specific for the detection of *S*. Enteritidis or *S*. Typhimurium without showing any cross-reaction with other *Salmonella* serovars or non-*Salmonella* species tested. The assay was further validated for its ability to detect *S.* Enteritidis in inoculated ground chicken, egg, whole milk, and cake mix in the presence of background natural microflora. A brief sample enrichment step also allows the resuscitation of stressed or injured cells before detection (Wu, 2008).

In the MaCIA platform, HCT-8 cells were used as a capture element instead of an antibody, which is traditionally used in a sandwich ELISA. In this study, HCT-8 cells out-performed the antibody (Figure 1C), and 30 min incubation was sufficient for optimal capture of viable bacteria by HCT-8 cells (Jaradat and Bhunia, 2003; Barrila et al., 2017) while 2 h was needed for sandwich ELISA. Improved bacterial capture by HCT-8 is attributed to the formation of a three-dimensional structure by mammalian cell monolayer (Figure 2E), creating a larger surface area for bacteria to bind. Furthermore, HCT-8 cell possesses surface receptor molecules for specific interaction with *Salmonella* adhesion factors. *S.* Enteritidis utilize type 1 fimbria to recognize and bind to high-mannose oligosaccharides, which are carried by various glycoproteins on the host cell surface (Kolenda et al., 2019). Long polar fimbriae also mediate adhesion of *Salmonella* to Peyer’s patches on the host cell (Bäumler et al., 1996). Besides, MaCIA was able to differentiate viable cells from dead *Salmonella* cells while sandwich immunoassay was unable. Lack of adhesion of dead *Salmonella* to HCT-8 may be due to the loss or denaturation of bacterial adhesins (Figure 2G). While in sandwich ELISA, bacterial surface antigens from dead cells were still able to bind the capture-antibody. MaCIA also showed strong signals when tested with stress-exposed *S*. Enteritidis cells suggesting a brief stress exposure (3 h) does not affect bacterial ability to interact with the HCT-8 cells (Figure 3) while such exposure caused a 20–48% reduction in ELISA signal for *Salmonella* in a previous study (Hahm and Bhunia, 2006).

The sensitivity of MaCIA was found to be about 1 × 10^6^ CFU/mL to 1 × 10^7^ CFU/mL, which is in agreement with a typical ELISA where antibodies serve as the capture molecule (Mansfield and Forsythe, 2000; Eriksson and Aspan, 2007) or ELISA with bacteriophage as a recognition molecule (Galikowska et al., 2011). However, MaCIA has the potential to outperform ELISA in some aspects, due to its ability to differentiate viable from dead bacteria. Viable pathogens that can adhere and invade into intestinal cells are of food safety concerns. MaCIA is a better choice over ELISA for the food industry when viable pathogens in food are the target. False-positive results generated by either ELISA or PCR due to the presence of non-viable pathogens could lead to unnecessary recalls, food waste, and economic loss. On the other hand, assays with higher sensitivity may be useful for detecting samples with low bacterial concentration, but enrichment is considered a necessary step to ensure accuracy (Bhunia, 2014). Assuming a 25 g sample contains 1 CFU of bacteria unless one performs a test on the entire 25 g sample, there is a high possibility that one would not be able to accurately detect the bacteria even with a sensor that has the sensitivity to detect 1 CFU. So, the sensitivity of an assay not only depends on the limit of detection but also on the sample size. Therefore, we proposed to perform MaCIA in concert with the traditional enrichment step, to offer a more reliable and accurate testing result.

The assay sensitivity was also affected by the food matrices tested. Ground chicken, raw eggs, whole milk, and cake mix were chosen since these products were implicated in *Salmonella* outbreaks, and they also represent foods with high protein, fat, or carbohydrate contents. In milk, the detection limit for *S*. Enteritidis was 1 × 10^5^ CFU/mL while in ground chicken, 1 × 10^6^ CFU/mL, in cake mix, 1 × 10^7^ CFU/mL, and in egg, 1 × 10^8^ CFU/mL (Figure 4C). Among the foods tested, eggs exhibited the highest interference while milk had the least. Egg contains about 13 g protein and 11 g fat per 100 g while whole milk contains only 3.15 g of protein and 3.25 g of fat per 100 g (Kuang et al., 2018).

Mammalian cell-based immunoassay is highly specific for *S*. Enteritidis and did not show any non-specific reaction with other *Salmonella* serovars, non-*Salmonella* organisms, or natural microflora present in uninoculated food samples. The specificity of MaCIA is attributed to the specificity of the reporter antibody, mAb-2F11 used, that binds the O-antigen (LPS) on the surface of *S.* Enteritidis (Masi and Zawistowski, 1995; Jaradat et al., 2004). The advantage of the MaCIA platform is that the specificity depends on the primary reporter antibody used. We have demonstrated that using a commercial anti-*Salmonella* mAb-F68C (Thermo-Fisher) as a reporter antibody, *Salmonella* enterica serovar Typhimurium can be detected on the MaCIA platform (Table 1). These results indicate that the MaCIA platform is versatile and can be adapted for a different target pathogen using an appropriate antibody.

The accuracy of MaCIA for *S*. Enteritidis was also confirmed by comparing the results with the reference methods, such as the FDA-BAM, USDA-FSIS, and PCR (Figure 6D). The three primer sets that were used in PCR (Supplementary Table 3) target *IE-1, IE-2* in *S.* Enteritidis, and *InvA* in *S.* Enteritidis and *S*. Typhimurium (Fratamico and Strobaugh, 1998; Wang and Yeh, 2002; Paião et al., 2013), which again confirm the accuracy of MaCIA for its ability to detect *S*. Enteritidis from spiked food samples.

The major advancement of the MaCIA is its extended shelf-life, at least for 14 weeks, that was achieved through formalin-fixation of HCT-8 cells. Formalin is routinely used to preserve tissues and cells and it protects protein from denaturation (Eltoum et al., 2001). Therefore, receptor molecules on formalin-fixed HCT-8 cells, remained active and enabled viable *Salmonella* binding without diminishing MaCIA’s performance. Previously, many attempts have been made to extend the shelf-life (functionality) of cells in CBB; however, none were satisfactory. Bhunia et al. (1995) used ultra-low temperature (freezing at −80 and −196°C) to extend the shelf-life of cells (up to 8 weeks) before performing the cytotoxicity assay for *L. monocytogenes*. However, the major drawback was the generation of high background signal originating from freeze-injured or dead mammalian cells. Banerjee et al. (2007) used modified growth conditions that included 5% fetal calf serum without any exogenous CO_2_ and was able to extend the viability of the lymphocyte cell line for 6–7 days at room temperature. Curtis et al. (2009) used an automated media delivery system integrated with a thermoelectric controller to keep endothelial cells healthy up to 16 weeks. More recently, Jiang et al. (2018) used a screen-printed hydrogel-encapsulated rat basophilic leukemia mast cell-based electrochemical sensor for the detection of quorum sensing molecules for fish spoilage and the sensor-generated stable signal for 10 days. However, these attempts required incorporating mammalian cells in a specially designed external device to ensure the success of detection.

## Conclusion

In conclusion, the present study demonstrates that MaCIA is a highly specific functional cell-based assay coupled with an immunoassay for the rapid and specific detection of the viable target pathogen. *S.* Enteritidis was used as a model pathogen which was successfully detected from food samples (ground chicken, shelled egg, whole milk, and cake mix) in 16–21 h using a conventional sample enrichment set up. The assay time (sample-to-result) was shortened to 10–12 h when an on-cell (on the MaCIA platform) sample enrichment was used. Thus, MaCIA could serve as a universal platform for other pathogens provided an appropriate cell line and a pathogen-specific antibody is used. The extended shelf-life of mammalian cells made MaCIA an attractive screening tool for point-of-need deployment. Furthermore, the MaCIA platform (24-well tissue culture plate) is suitable for testing at least 10 samples (plus positive and negative controls) in duplicate on a single plate thus reducing overall cost per sample testing.

## Materials and Methods

### Mammalian Cell Culture

HCT-8 cell line (ATCC, Manassas, VA, United States) was maintained in Dulbecco’s modified Eagles medium (DMEM; Thermo Fisher Scientific) with 10% fetal bovine serum (FBS; Bio-Techne Sales Corp, Minneapolis, MN, United States) at 37°C with 5% CO_2_ in cell culture flasks (T25). For all experiments, HCT-8 cells were seeded in 24-well tissue culture plates (Fisher Scientific) at a density of 5 × 10^4^ cell/mL/well. Media were replaced on day 4 and a final cell density of 2 × 10^5^ cell/mL (monolayer) was achieved on day 5. Cell monolayers were washed twice with PBS (0.1 M, pH 7.0) and used immediately (Live HCT-8 cell assay) or exposed to 4% formaldehyde (Polysciences Inc., Warrington, PA) of 500 μL/well and incubated at room temperature for 10 min (Formalin-fixed HCT-8). Formaldehyde solution was removed and the cell monolayers were washed three times with PBS. Formalin-fixed cells were stored in 1 mL PBS/well for 14 weeks at 4°C or until use.

### Bacterial Culture and Growth Media

Bacterial strains (Table 1) were stored as 10% glycerol stocks at −80°C. To revive frozen cultures, each strain was streaked onto tryptic soy agar (TSA; Thermo Fisher Scientific, Rochester, NY, United States) plate and incubated at 37°C for 18 h to obtain pure colonies. A single colony of each strain was inoculated and propagated in tryptic soy broth containing 0.5% yeast extract (TSBYE; Thermo Fisher Scientific) at 37°C for 18 h with shaking at 120 rpm.

### Development and Specificity of MaCIA

HCT-8 cell monolayers were prepared and maintained as described above in 24-well plates. Overnight grown bacterial cultures (Table 1) were diluted in PBS to achieve a cell concentration of 1 × 10^7^ CFU/ml. To obtain dead cells, cell suspensions were treated with heat (80°C for 10 min) or formaldehyde (4% for 10 min) and plated on TSA to ensure bacterial inactivation. One milliliter of bacterial cell suspensions was added into each well containing HCT-8 cells and incubated for 30 min at 37°C (Jaradat and Bhunia, 2003; Barrila et al., 2017). Cell monolayers (live or formalin-fixed) were washed 2–3 times with PBS gently and sequentially probed with either mAb-2F11 (3.06 μg/mL) (Jaradat et al., 2004) or mAb-F68C (0.2 μg/mL; Catalog # MA1-7443; Thermo Fisher Scientific) as primary antibodies, and anti-mouse HRP-conjugated IgG (0.1 μg/mL; Cell-Signaling, Danvers, MA, United States) as secondary antibodies for 1.5 h each at room temperature. Both antibodies were suspended in PBS containing 3% bovine serum albumin (BSA; Sigma-Aldrich). For one-step antibody probing, both mAb-2F11 and anti-mouse HRP conjugated secondary antibodies were mixed in PBS containing 3% BSA and incubated for 1.5 h. Cell monolayers were washed 3 times with PBS and the color was developed by adding 500 μl/well substrate solution (o-phenylenediamine, OPD) containing hydrogen-peroxide; Sigma-Aldrich). The oxidative coupling of OPD to 2,3-diaminophenazine, an orange-brown substance, was catalyzed by HRP at room temperature in the dark for 10 min. The intensity of the colored product was measured using a microplate reader (BioTek, Winooski, VT, United States) at a wavelength of 450 nm.

### Sandwich ELISA

High-affinity (4HBX) ELISA plates (Thermo Fisher Scientific) were coated with mAb-2F11 for 2 h at 37°C, followed by 3 times wash with PBS-T (PBS containing 0.01% Tween-20). Freshly prepared BSA-PBS solution (1 mg/mL) was used for blocking at 4°C overnight, followed by 2 × PBS-T wash. Freshly prepared viable or formalin-inactivated cells of *S*. Enteritidis (1 × 10^8^ cells/100 μl) were added to each well and incubated at 37°C for 30 min or 2 h. Anti- *Salmonella* pAb-3288 (2.86 μg/mL) used as a reporter (Abdelhaseib et al., 2016) and an HRP-conjugated anti-rabbit antibody (0.25 μg/mL) as the secondary antibody. After 3 washes with PBS-T, the OPD substrate was added and the absorbance (450 nm) was measured.

### Western Blot

The whole-cell lysate of *L. monocytogenes* F4244, *P. aeruginosa* PRI99, *E. coli* EDL933, and *S.* Enteritidis PT21 overnight cultures (5 mL each) was prepared by sonication (Branson, Danbury, CT, United States). Bacterial samples were sonicated in an ice bucket (three 10 s cycles at 30-s intervals) and centrifuged for 10 min at 14,000 rpm (Eppendorf) at 4°C to separate the soluble fraction (supernatant) from the bacterial debris (pellet). The protein concentration was determined by the BCA method (Thermo Fisher Scientific). Equal amounts of proteins were separated on SDS-PAGE gel (10% polyacrylamide) and electro-transferred to polyvinylidene difluoride (PVDF) membrane (Fisher Scientific) (Singh et al., 2016; Drolia et al., 2018). Primary and secondary antibodies were diluted as above. Membranes were first probed with mAb-2F11 at 4°C overnight, and then with anti-mouse HRP conjugated antibody at room temperature for 1.5 h. LumiGLO reagent (Cell-Signaling Technology) was used to visualize the bands using the Chemi-Doc XRS system (Bio-Rad).

### Immunofluorescence and Giemsa Staining

After exposure of formalin-fixed HCT-8 cell monolayers to viable or dead *S.* Enteritidis (1 × 10^8^ cells/ml) for 30-min, the wells of the chambered slides (Fisher Scientific) were washed with PBS to remove unattached bacterial cells (as above). After immunoprobing with mAb-2F11, the monolayers were washed and probed with Alexa Fluor 488 conjugated anti-mouse antibody for 1.5 h at room temperature in the dark, followed by three PBS wash. Note, antibody concentrations used were the same as above. The monolayers were counterstained with DAPI (500 ng/mL; Cell-Signaling) for nuclear staining and the slides were mounted using an antifade reagent (Cell-Signaling). Images were acquired using the Nikon A1R confocal microscope with a Plano AP VC oil immersion objective (Drolia et al., 2018) and were processed with the Nikon Elements software at the Purdue Bindley Bioscience Imaging Facility.

For Giemsa staining, the formalin-fixed HCT-8 cell monolayers were exposed to viable or dead *S.* Enteritidis cells as above, air-dried, and immersed in Giemsa staining solution for 20 min. Giemsa staining solution was prepared using a 20-fold dilution of the KaryoMAX Giemsa staining solution (Thermo-Fisher) in deionized water. The slides were examined under a Leica DAS Microscope at the magnification of 1,000×.

### Sensitivity of MaCIA

HCT-8 cell monolayers were prepared and maintained as described above in 24-well tissue culture plates. Overnight grown fresh *S.* Enteritidis PT21 culture was serially diluted to obtain 1 × 10^8^ CFU/mL to 1 × 10^4^ CFU/mL using PBS or homogenized 25 g food samples (Supplementary Table 2) in 225 mL BPW (Becton Dickinson, Sparks, MD, United States). One milliliter of each diluted sample was added onto HCT-8 cell monolayer and was incubated at 37°C for 30 min. The remaining steps were the same as above.

### Detection of Stressed Cells Using MaCIA

Freshly prepared *S.* Enteritidis cells (2.17 × 10^8^ CFU/ml) suspended in TSB were exposed to cold (4°C), heat (45°C), acidified TSB (pH, 4.5) and 5.5% NaCl for 3 h, as reported before (Hahm and Bhunia, 2006). Bacterial cells were washed with PBS and added onto the fixed HCT-8 monolayer for 30-min and probed with mAb-2F11 as above.

### *Salmonella* Growth Kinetics Assessment

Overnight-grown *S.* Enteritidis PT21 cultures were serially diluted in PBS to achieve a concentration of 1 × 10^2^ CFU/mL. One hundred microliters of the diluted culture were added into 25 g of each ground chicken, whole fat milk, liquid eggs, and cake mix with 225 mL BPW and were incubated at 4°C for 24 h. The samples were then incubated at 37°C for 20 h with shaking at 120 rpm and enumerated on XLD (xylose lysine deoxycholate) agar plates (Remel, San Diego, CA) at every hour. *S.* Enteritidis counts in artificially inoculated samples at earlier stages of growth was determined by directly plating 1, 0.5, 0.1 mL of the sample on XLD plates with four repeats (1, 2, and 3 h); and *S.* Enteritidis counts from the later stages of growth (3 h and after) was obtained after serially diluting the samples in PBS. The growth of *S.* Enteritidis in food samples enriched using BPW was modeled using the Gompertz equation (Silk et al., 2002; Kim and Bhunia, 2008) through Prism software version 8.0. Lag-phase duration (LPD) and exponential growth rate (EGR) were calculated from the Gompertz model and were used to determine an enrichment time required for each food product to reach an optimum *S.* Enteritidis concentration required for detection by MaCIA, assuming the initial concentration was 1 CFU/25 g of sample.

### Food Sample Testing With MaCIA and Validation With the FDA and USDA Methods

Food samples (ground poultry, milk, egg, or cake mix) were inoculated with variable concentrations of *S.* Enteritidis PT21. To simulate cold storage, inoculated foods were stored at 4°C for 24 h. Samples (25 g in 225 mL BPW) were then homogenized or pummeled using hands and incubated at 37°C for 14–19 h (Supplementary Table 1) with shaking at 120 rpm. One milliliter of enriched food sample was added into each well of MaCIA for 30 min, followed by immunoprobing as above.

For direct on-cell enrichment, the homogenized food suspensions (1 ml of each food sample) were dispensed into wells containing formalin-fixed HCT-8 cells (MaCIA) and incubated for 7–9 h. After the removal of food samples, wells were washed 3 times with PBS before immunoprobing and color development. *Salmonella* counts in enriched food samples (inoculated or uninoculated) were enumerated on XLD plates. The presence of background bacteria in uninoculated food samples was assessed on TSA plates after incubation at 37°C for 24 h. For the blind test, the inoculation of the samples was performed by XB, while the MaCIA test was done by LX in a blinded fashion without prior knowledge of samples that were inoculated with *Salmonella*.

Inoculated food samples were also analyzed by the FDA-BAM (FDA, 2001) or USDA-FSIS (2013) method as before. The ground chicken was processed according to the USDA-FSIS method, while shelled egg, whole milk, and cake mix were prepared based on the FDA-BAM. Twenty-five gram of each prepared sample was then enriched in 225 mL of BPW (ground chicken), trypticase soy broth (shelled egg), and lactose broth (whole milk and cake mix) at 37°C for 24 h followed by sequential enrichment in RV (Rappaport-Vassiliadis) broth and TT (tetrathionate) broth at 42°C for 24 h. Samples were then plated on selective BGS (Brilliant Green Agar with Sulfadiazine) or XLD agar plates to isolate colonies, which were further confirmed by PCR assay.

For PCR assay, DNA was extracted from the isolated colonies by the boiling method (Kim and Bhunia, 2008; Kim et al., 2015). The primer sequences and the putative product sizes for each amplicon are listed in Supplementary Table 3 (Wang and Yeh, 2002; Paião et al., 2013). PCR reaction mixture (25 μL) contained 1 μL of DNA template, 0.2 μM of each primer, 2.5 mM MgCl_2_, 200 μM of dNTP, 1 x GoTaq Flexi buffer of buffer and 1 U of GoTaq Flexi DNA polymerase (Promega) (Singh et al., 2014). The PCR amplification was performed in the Proflex PCR system with an initial denaturation at 94°C for 3 min, 35 amplification cycles consisting of 1 min of denaturation at 94°C, 1.5 min of annealing at 50°C, and 1.5 min of elongation at 72°C. DNA amplicons were analyzed using agarose gel (1.5%, wt/vol) electrophoresis containing ethidium bromide (0.5 μg of/mL).

### Swab Sample Testing

Chicken thigh cuts (procured from a local grocery store) were inoculated with overnight grown *S.* Enteritidis PT21 at 1.35 × 10^3^ to 1.35 × 10^5^ CFU per 50 cm^2^ evenly on the skin of chicken thighs. Inoculated samples were stored at 4°C for 24 h. BPW-soaked sterile rayon tipped swab applicators (Puritan, Guilford, ME, United States) were used to swab the chicken skin and were vortexed in 1.1 mL of BPW. One milliliter of the sample was added into each well of MaCIA and incubated at 37°C for 7 h for on-cell enrichment, followed by immunoprobing as above. The rest of the swabbed sample (0.1 mL) was used to enumerate *Salmonella* on XLD plates.

### Statistical Analysis

All data were analyzed using GraphPad Prism software (San Diego, CA, United States). The unpaired *t*-test was used when comparing two datasets. Tukey’s multiple comparison test was also used when comparing more than two datasets. All data were presented with mean ± standard error of the mean (SEM).

## Data Availability Statement

The original contributions presented in the study are included in the article/Supplementary Material, further inquiries can be directed to the corresponding author.

## Author Contributions

LX, XB, RD, and AB conceived and designed the experiments. LX, XB, ST, YL, and RD performed the experiments. LX, XB, and AB analyzed the data. LX and AB wrote the manuscript. All authors approved the final version.

## Conflict of Interest

The authors declare that the research was conducted in the absence of any commercial or financial relationships that could be construed as a potential conflict of interest.

## Supplementary Material

Supplementary Figure 1 Detection sensitivity of MaCIA tested against the different concentrations of *S. enterica* serovar Enteritidis cells suspended in ground chicken slurry (in buffered peptone water). D, dead cells.

Supplementary Figure 2 Experimental set-up of the blind test using an on-cell (MaCIA) enrichment method. **(A,B)** The checkerboard filled areas correspond with sample a; the diagonal stripes filled areas correspond with sample b; No pattern-filled area corresponds with negative control for each food product. The numbers in the table represent the concentration (CFU/mL) of the inoculant, *S.* Enteritidis PT21. **(C)** Blinded test using on-cell enrichment. Positive samples were inoculated with 25 CFU/mL cold-stored *S.* Enteritidis PT21. Neg: negative control. a, b: blind tested samples.

Supplementary Figure 3 Tryptic soy agar (TSA) plates showing the presence of background bacterial populations from different food samples except for the eggs.

Supplementary Table 1 Proposed enrichment time for different food products before testing with MaCIA.

Supplementary Table 2 Total detection time required for each method.

Supplementary Table 3 PCR primer sequences used.
