# Supplementary figures and images for: Mammalian Cell-Based Immunoassay for Detection of Viable Bacterial Pathogens
Source: Front Microbiol. 2020 Nov 23;11:575615. doi: 10.3389/fmicb.2020.575615 (PMC7732435; doi:10.3389/fmicb.2020.575615)

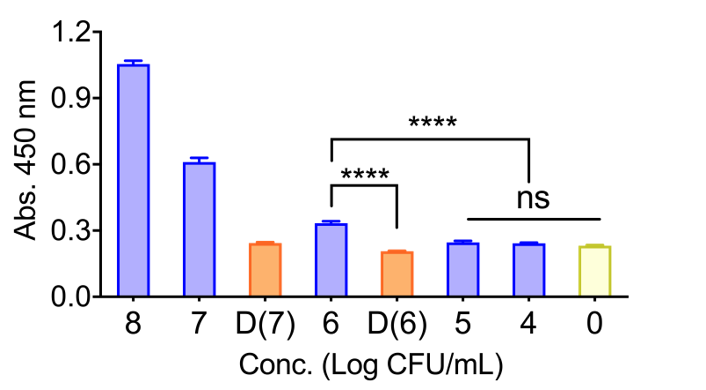

Supplement: Supplementary Figure 1 — Detection sensitivity of MaCIA tested against the different concentrations of S. enterica serovar Enteritidis cells suspended in ground chicken slurry (in buffered peptone water). D, dead cells. [file Image_1.TIF]

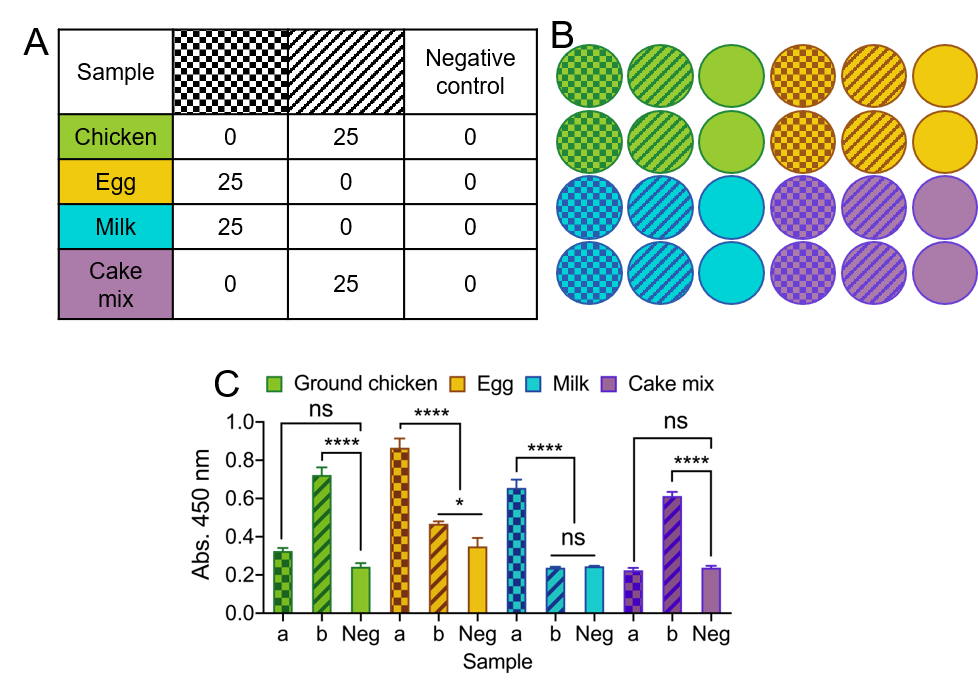

Supplement: Supplementary Figure 2 — Experimental set-up of the blind test using an on-cell (MaCIA) enrichment method. (A,B) The checkerboard filled areas correspond with sample a; the diagonal stripes filled areas correspond with sample b; No pattern-filled area corresponds with negative control for each food product. The numbers in the table represent the concentration (CFU/mL) of the inoculant, S. Enteritidis PT21. (C) Blinded test using on-cell enrichment. Positive samples were inoculated with 25 CFU/mL cold-stored S. Enteritidis PT21. Neg: negative control. a, b: blind tested samples. [file Image_2.TIF]

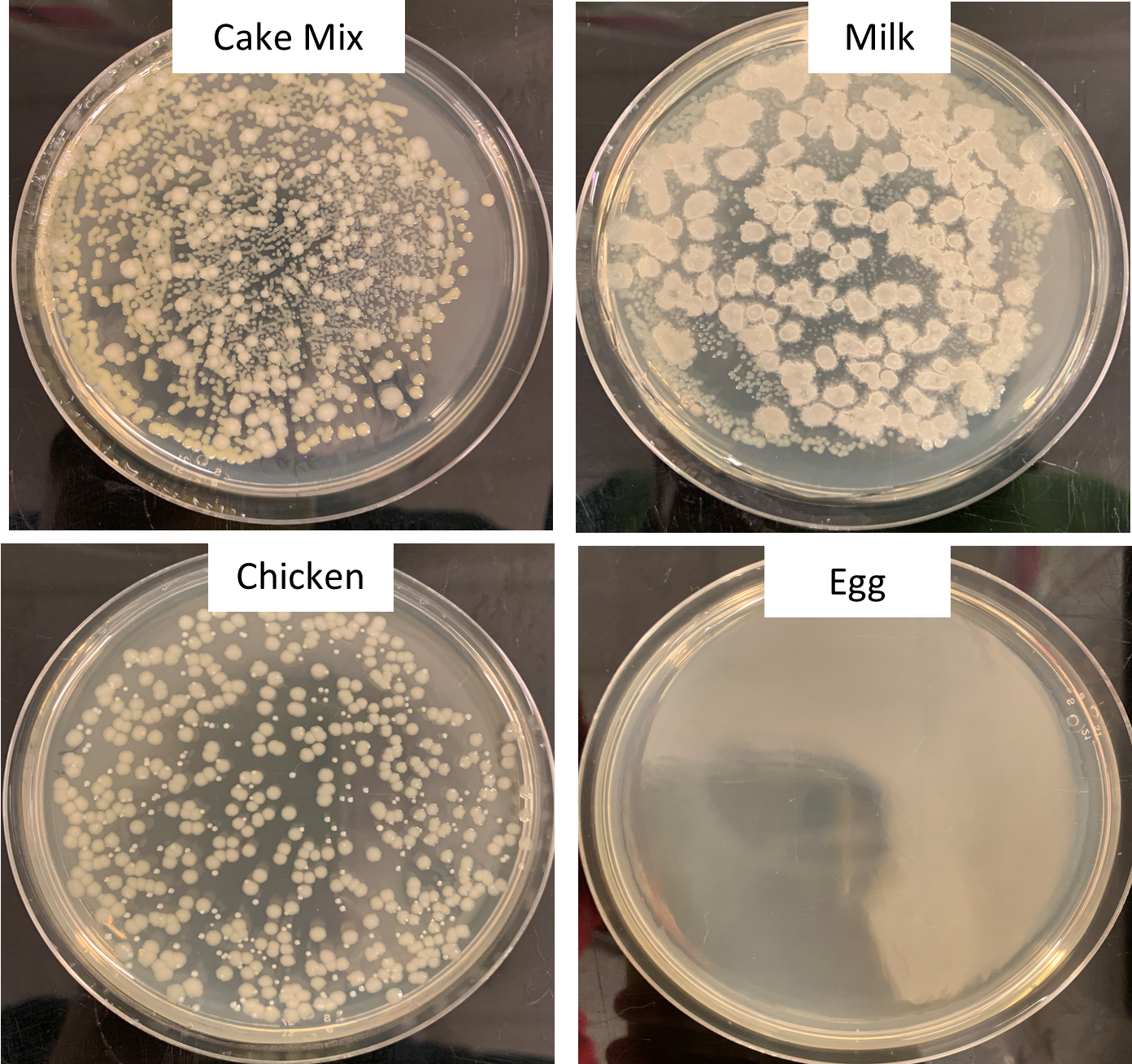

Supplement: Supplementary Figure 3 — Tryptic soy agar (TSA) plates showing the presence of background bacterial populations from different food samples except for the eggs. [file Image_3.TIF]
