# Supplementary material for: Mammalian Cell-Based Immunoassay for Detection of Viable Bacterial Pathogens
Source: Front Microbiol. 2020 Nov 23;11:575615. doi: 10.3389/fmicb.2020.575615 (PMC7732435; doi:10.3389/fmicb.2020.575615)
Supplement: Supplementary Table 1 — Proposed enrichment time for different food products before testing with MaCIA. [file Table_1.DOCX]

**Table S1.** Proposed enrichment time for different food products before testing with MaCIA

| Food sample | Lag phase duration (LPD) (h) | Exponential growth rate (EGR)  (Log (CFU/mL)/h) | Proposed enrichment time (h) | MaCIA detection time using on-cell enrichment (h) |
| --- | --- | --- | --- | --- |
| Ground chicken | 2.204±0.130 | 0.896±0.019 | 14 | 7 |
| Shelled eggs | 2.319±0.100 | 0.934±0.016 | 19 | 7 |
| Whole milk | 2.427±0.110 | 0.767±0.013 | 16 | 9 |
| Cake mix | 2.260±0.710 | 0.983±0.133 | 16 | 9 |
