# Supplementary material for: Mammalian Cell-Based Immunoassay for Detection of Viable Bacterial Pathogens
Source: Front Microbiol. 2020 Nov 23;11:575615. doi: 10.3389/fmicb.2020.575615 (PMC7732435; doi:10.3389/fmicb.2020.575615)
Supplement: Supplementary Table 2 — Total detection time required for each method. [file Table_2.DOCX]

**Table S2.** Total detection time required for each method

| Test sample | Assay completion time (hour) | | | |
| --- | --- | --- | --- | --- |
|  | MaCIA  (Out-cell enrichment) | MaCIA  (On-cell Enrichment) | USDA-FSIS | FDA-BAM |
| Ground Chicken | 16 | 10 | 72 | NA |
| Shelled egg | 21 | 10 | NA | 168 |
| Whole milk | 18 | 12 | NA | 72 |
| Cake mix | 18 | 12 | NA | 72 |

NA, not applicable
