# Supplementary material for: Mammalian Cell-Based Immunoassay for Detection of Viable Bacterial Pathogens
Source: Front Microbiol. 2020 Nov 23;11:575615. doi: 10.3389/fmicb.2020.575615 (PMC7732435; doi:10.3389/fmicb.2020.575615)
Supplement: Supplementary Table 3 — PCR primer sequences used. [file Table_3.DOCX]

**Table S3.** PCR primer sequences used

| **Pathogen** | **Target gene** | **Primer** | **Sequence (5'-3')** | **Product Size (bp)** | **References** |
| --- | --- | --- | --- | --- | --- |
| *Salmonella enterica* serovar Enteritidis | Insertion element  (IE-1) | F | AGT GCC ATA CTT TTA ATG AC | 316 | (Fratamico and Strobaugh, 1998; Wang and Yeh, 2002) |
|  |  | R | ACT ATG TCG ATA CGG TGG G |  |  |
|  | Insertion element  (IE-2) | F | GGA TAA GGG ATC GAT AAT TGC | 559 | (Wang and Yeh, 2002) |
|  |  | R | GGA CTT CCA GTT ATA GTA GG |  |  |
| *Salmonella enterica* serovar Enteritidis and Typhimurium | Invasion protein A (Inv-A) | F | CGG TGG TTT TAA GCG TAC TCT T | 796 | (Fratamico and Strobaugh, 1998; Paião et al., 2013) |
|  |  | R | CGA ATA TGC TCC ACA AGG TTA |  |  |

**References**

Fratamico, P.M., and Strobaugh, T.P. (1998). Simultaneous detection of *Salmonella* spp and *Escherichia coli* O157:H7 by multiplex PCR. *J. Indust. Microbiol. Biotechnol.* 21**,** 92-98.

Paião, F.G., Arisitides, L.G.A., Murate, L.S., Vilas-Bôas, G.T., Vilas-Boas, L.A., and Shimokomaki, M. (2013). Detection of *Salmonella* spp, *Salmonella* Enteritidis and Typhimurium in naturally infected broiler chickens by a multiplex PCR-based assay. *Braz. J. Microbiol.* 44**,** 37-42.

Wang, S.J., and Yeh, D.B. (2002). Designing of polymerase chain reaction primers for the detection of *Salmonella* Enteritidis in foods and faecal samples. *Lett. Appl. Microbiol.* 34**,** 422-427.
